# Competition Between Strains of *Borrelia afzelii* in Immature *Ixodes ricinus* Ticks Is Not Affected by Season

**DOI:** 10.3389/fcimb.2019.00431

**Published:** 2019-12-19

**Authors:** Dolores Genné, Anouk Sarr, Olivier Rais, Maarten J. Voordouw

**Affiliations:** ^1^Laboratory of Ecology and Evolution of Parasites, Institute of Biology, University of Neuchâtel, Neuchâtel, Switzerland; ^2^Laboratory of Ecology and Epidemiology of Parasites, Institute of Biology, University of Neuchâtel, Neuchâtel, Switzerland; ^3^Department of Veterinary Microbiology, Western College of Veterinary Medicine, University of Saskatchewan, Saskatoon, SK, Canada

**Keywords:** *Borrelia afzelii*, co-infection, inter-strain competition, *Ixodes ricinus*, transmission, vector-borne pathogen, Lyme disease

## Abstract

Vector-borne pathogens often consist of genetically distinct strains that can establish co-infections in the vertebrate host and the arthropod vector. Co-infections (or mixed infections) can result in competitive interactions between strains with important consequences for strain abundance and transmission. Here we used the spirochete bacterium, *Borrelia afzelii*, as a model system to investigate the interactions between strains inside its tick vector, *Ixodes ricinus*. Larvae were fed on mice infected with either one or two strains of *B. afzelii*. Engorged larvae were allowed to molt into nymphs that were subsequently exposed to three seasonal treatments (artificial summer, artificial winter, and natural winter), which differed in temperature and light conditions. We used strain-specific qPCRs to quantify the presence and abundance of each strain in the immature ticks. Co-infection in the mice reduced host-to-tick transmission to larval ticks and this effect was maintained in the resultant nymphs at 1 and 4 months after the larva-to-nymph molt. Competition between strains in co-infected ticks reduced the abundance of both strains. This inter-strain competition occurred in the three life stages that we investigated: engorged larvae, recently molted nymphs, and overwintered nymphs. The abundance of *B. afzelii* in the nymphs declined by 40.5% over a period of 3 months, but this phenomenon was not influenced by the seasonal treatment. Future studies should investigate whether inter-strain competition in the tick influences the subsequent strain-specific transmission success from the tick to the vertebrate host.

## Introduction

Many infections consist of multiple strains or genotypes of the same pathogen, which are called co-infections (Read and Taylor, 2001; Balmer and Tanner, 2011; Alizon et al., 2013). Co-infections (also referred to as mixed infections or multiple-strain infections) will result in positive or negative interactions between co-infecting strains that result in facilitation (Taylor et al., 1997; de Roode et al., 2004; Abkallo et al., 2015) or competition (Bruce et al., 2000; de Roode et al., 2005a,b; Bell et al., 2006; Harrison et al., 2006; Mideo, 2009; Pollitt et al., 2011; Khan et al., 2019), respectively. In facilitation (positive interaction), the performance (e.g., transmission, fitness, abundance) of a particular strain in a mixed infection is enhanced compared to when this strain infects the host by itself. In competition (negative interaction), the performance of a particular strain in a mixed infection is reduced compared to the single-strain infection. Multiple-strain infections are important because interactions between strains in their host shape the optimal life history strategies of pathogen transmission and virulence, which is the level of harm that the pathogen causes in its host (Mideo, 2009; Alizon et al., 2013).

In the case of vector-borne pathogens, mixed infections can occur in both the vertebrate host and the arthropod vector. Numerous studies have investigated interactions between pathogen strains in the vertebrate host (Bruce et al., 2000; de Roode et al., 2005a; Bell et al., 2006; Harrison et al., 2006; Pollitt et al., 2011; Reif et al., 2014). In contrast, studies on inter-strain interactions in the arthropod vector are rare (Reif et al., 2014; Pollitt et al., 2015; Genné et al., 2018), but these studies have shown that these interactions exist and that they influence the performance of the pathogen strains inside the arthropod vector. The population dynamics of vector-borne pathogens inside their arthropod vectors are also highly dependent on abiotic factors, such as temperature (Sternberg and Thomas, 2014). For example, warmer temperatures reduce the vectorial capacity of malaria mosquitoes (Paaijmans et al., 2011) and high temperatures can clear the Lyme disease pathogen from ticks so that they are no longer infectious to mice (Shih et al., 1995). Taken together, these observations suggest that temperature (and other abiotic variables) could influence the outcome of the interactions between strains in mixed infections in the arthropod vector, but to date there are no studies on this subject.

In the present study, we used the tick-borne spirochete, *Borrelia afzelii*, to test whether season (temperature and light) influences the competition between strains in mixed infections inside its long-lived tick vector. *B. afzelii* is the most common cause of Lyme borreliosis in Europe and is transmitted among small mammal reservoir hosts by the hard tick *Ixodes ricinus* (van Duijvendijk et al., 2015). Larvae acquire *B. afzelii* when they feed on an infected host [there is no transovarial transmission (Richter et al., 2012; Rollend et al., 2013)] and the engorged larvae molt into flat (unfed) nymphs by early fall. These flat nymphs overwinter in the soil (Dusbabek et al., 1971; Daniel et al., 1972) and enter a diapause phase (Belozerov, 1982; Dautel et al., 2008; Gray et al., 2016). The nymphs become active the following spring and search for new hosts (Belozerov, 2009; Gray et al., 2016). From a life history perspective, the nymphs are the most important stage for tick-to-host transmission because they feed on competent reservoir hosts and because their density is an order of magnitude greater than adult ticks (Kurtenbach et al., 2006; Tsao, 2009).

In areas where Lyme borreliosis is endemic, mixed infections in the nymph are common (Qiu et al., 2002; Pérez et al., 2011; Durand et al., 2015, 2017). A recent experimental infection study found that strains of *B. afzelii* experience competition inside *I. ricinus* nymphs (Genné et al., 2018). The population size of *B. afzelii* inside the unfed nymph decreases over time suggesting that competition between strains could intensify with increasing nymphal age (Jacquet et al., 2017; Pospisilova et al., 2019). There is indirect evidence that spirochete load in the nymph is an important phenotype for nymph-to-host transmission: *B. afzelii* strains with higher population sizes in nymphs are more common in the field (Durand et al., 2017). In summary, Lyme disease is an interesting system for studying whether inter-strain competition changes over the life cycle of a long-lived arthropod vector and whether this competition is influenced by abiotic factors like temperature.

We recently used an experimental infection approach to show that strains of *B. afzelii* experience competition inside *I. ricinus* nymphs that were 1 month old (Genné et al., 2018). As part of that study, we also collected engorged larvae, and we allowed a subset of nymphs to age under different seasonal treatments (i.e., different temperature and light conditions that represented summer vs. winter), but these ticks were not analyzed until now. The aim of the present study was to investigate whether competition between strains of *B. afzelii* occurs at different stages of the life cycle of immature *I. ricinus* ticks, and whether this competition is influenced by seasonal treatment. We made three predictions with respect to the interactions between interstrain competition, life cycle stage, and seasonal treatment. First, competition between strains would occur at each of the three life cycle stages: engorged larvae, 1-month-old nymphs, and 4-month-old nymphs. Second, the nymphal spirochete load would decrease with nymphal age and that competition between strains would be more intense in the older nymphs. Third, the nymphal spirochete load would decrease quickly under summer conditions but remain static under winter conditions.

## Materials and Methods

### General

In a previous study, we investigated competition between two strains of *B. afzelii* (Fin-Jyv-A3 and NE4049) in the rodent host *Mus musculus* and in the tick vector *I. ricinus* (Genné et al., 2018). We tested the effects of interstrain competition on two phenotypes of *B. afzelii*: strain-specific host-to-tick transmission and strain-specific spirochete load in ticks. Both of these phenotypes were measured in 1-month-old nymphs (*n* = 301) that had fed as larvae on experimentally infected mice, and that had been killed 1 month after the larva-to-nymph molt. What is new in the present study is that we investigated competition in the engorged larvae immediately following drop-off (*n* = 142), and in 4-month-old nymphs that were allowed to age under different seasonal treatments (*n* = 357). These two additional tick age classes allowed us to investigate competition between strains of *B. afzelii* at three different time points of the tick life cycle.

### Strains of *B. afzelii* and Mice

*Borrelia afzelii* strains NE4049 and Fin-Jyv-A3 were chosen for this study because both strains are highly infectious to rodent hosts (Genné et al., 2018). Strains NE4049 and Fin-Jyv-A3 were obtained from an *I. ricinus* nymph in Neuchatel, Switzerland and from a bank vole (*Myodes glareolus*) in Jyväskylä, Finland, respectively. Both strains were passaged fewer than five times to avoid the loss of the plasmids that carry the virulence genes that are critical for infection (Tonetti et al., 2015; Cayol et al., 2018). These strains have strain ID numbers 1961 and 1887 in the *Borrelia* multi-locus sequence type (MLST) database. Strain NE4049 has MLST 679 and *ospC* allele A10 and strain Fin-Jyv-A3 has MSLT 676 and *ospC* allele A3. The two *ospC* alleles used in this study (A3 and A10) have a genetic distance of 23.19% and an amino acid distance of 62.57%. The concatenated sequences of these two MLSTs differ at 9 base pairs over 4,785 bp (i.e., they are 99.81% similar).

**Figure 1:**
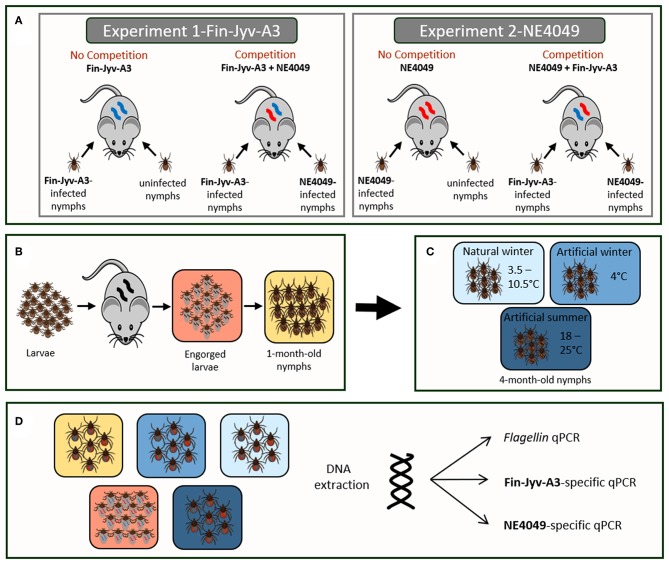
**(A)** The study was divided into two competition experiments that differed in the focal strain. In experiments 1 and 2, the focal strain was Fin-Jyv-A3 and NE4049, respectively. Each experiment was divided into two groups: no competition (infection with the focal strain only) and competition (infection with both strains). The sample size of each group was 10 mice. In the no competition treatment, each mouse was infected with 5 nymphs infected with the focal strain and 5 uninfected nymphs. In the competition treatment, each mouse was infected with 5 nymphs infected with strain Fin-Jyv-A3 and 5 nymphs infected with strain NE4049. **(B)** At 34 days post-infection, the mice were infested with ~100 larvae. Engorged larvae were allowed to molt into nymphs under laboratory conditions. **(C)** One month after the larva-to-nymph molt, the 1-month-old nymphs were exposed for 3 months to one of the three seasonal treatments: natural winter, artificial winter, and artificial summer. **(D)** Ticks were sacrificed at three different ages: engorged larvae, 1-month-old nymphs, and 4-month old nymphs. Only the 4-month old nymphs had been exposed to three different seasonal treatments (natural winter, artificial winter, and artificial summer). DNA was extracted from all ticks and the total number of spirochetes in each tick was estimated using *flagellin* qPCR. For the co-infected ticks, the abundance of strain Fin-Jyv-A3 and strain NE4049 was estimated using a strain-specific qPCR that targeted the *ospC* A3 allele and the *ospC* A10 allele, respectively.

The details of this experimental infection study were described in Genné et al. (2018) and are shown in Figure 1. Briefly, we performed two independent experiments to test whether the presence of a competitor strain influenced the performance of the focal strain. In experiments 1 and 2, the focal strain was Fin-Jyv-A3 and NE4049, respectively. Each experiment contained two infection treatments: single infection with the focal strain and co-infection with both strains. Thus, 40 female *Mus musculus* BALB/c mice aged 5 weeks were randomly assigned to four different experimental infection groups (*n* = 10 per group): Fin-Jyv-A3 (Single infection), Fin-Jyv-A3 + NE4049 (Co-infection), NE4049 (Single infection), and NE4049 + Fin-Jyv-A3 (Co-infection). Mice were infected with the appropriate strains of *B. afzelii* via the bite of experimentally infected *I. ricinus* nymphs.

Mice in the co-infection treatment (Figure 1A), were infested with 5 nymphs putatively infected with strain Fin-Jyv-A3 and 5 nymphs putatively infected with strain NE4049 (total of 10 nymphs). Mice in the single strain infection treatments were infested with 5 nymphs putatively infected with the focal strain and 5 uninfected nymphs (total of 10 nymphs). Prior to the nymphal infestation, we determined the prevalence of infection by testing a random sample of Fin-Jyv-A3 nymphs (*n* = 10) and NE4049 nymphs (*n* = 14) using the *flagellin* qPCR. The prevalence of infection was 70.0% (7/10) for the Fin-Jyv-A3 nymphs and 71.4% (10/14) for the NE4049 nymphs. Thus, the expected number of infected nymphs to which the mice were exposed was 3.50 for strain Fin-Jyv-A3 and 3.57 for strain NE4049. The mean spirochete load in the subset of infected nymphs was ~380 for the Fin-Jyv-A3 nymphs and ~198 for the NE4049 nymphs. Assuming that the number of spirochetes inoculated by a nymph into a mouse is linearly related to the product of the number of infected nymphs and the mean spirochete load per infected nymph, we calculate that the infectious dose for strain Fin-Jyv-A3 (3.57 infected nymphs*380 spirochetes/nymph = 1,356) was almost two times larger than the infectious dose for strain NE4049 (3.50 infected nymphs*198 spirochetes/nymph = 694 spirochetes).

### Age Classes of the Ticks

At 34 days post-infection, each infected mouse was infested with ~100 pathogen-free *I. ricinus* larvae (Figure 1B). Following drop-off, ~50 engorged larvae were collected from each mouse and stored in individual Eppendorf tubes to facilitate random sampling. Each Eppendorf tube contained a piece of moistened paper towel to maintain high humidity. The engorged larvae were randomly assigned to be sacrificed at one of three ages: engorged larvae, 1-month-old nymphs, and 4-month-old nymphs. Immediately following drop-off, up to 6 engorged larvae per mouse were frozen at −20°C for the engorged larva age class. The remaining engorged larvae were allowed to molt into nymphs under standard laboratory conditions. At 1 month after the larva-to-nymph molt (22 January 2016), up to 10 nymphs per mouse were frozen at −20°C for the 1-month-old nymph age class. The remaining nymphs were randomly assigned to three different seasonal treatments (see below). At 4 months after the larva-to-nymph molt (16 April 2016), up to 15 nymphs per mouse were frozen at −20°C for the 4-month-old nymph age class. The tick age classes, mouse sample sizes, and tick sample sizes are shown in Table 1.

**Table 1:** Sample sizes are shown for immature *I. ricinus* ticks that were collected in our previous study (1-month-old nymphs) and the present study (larvae and 4-month-old nymphs).

**Stage**	**Age**	**Seasonal treatment**	**Infected mice**	**Ticks per mouse**	**Total ticks**
Larva	2 days	NA	33	5–6	142
Nymph	1 month	NA	33	10	301
Nymph	4 months	Combined	26	15	357
Nymph	4 months	Phytotron	26	5	119
Nymph	4 months	Fridge	26	5	120
Nymph	4 months	Underground	26	5	118

*Ticks that had fed as larvae on mice infected with one or two strains of B. afzelii were sacrificed at three different ages: 2 days after drop-off, 1 month after the larva-to-nymph molt, and 4 months after the larva-to-nymph molt, which corresponds to two different stages: larva and nymph. The 4-month-old nymphs had been exposed to three different seasonal treatments: artificial summer (phytotron), artificial winter (fridge), and natural winter (underground). For each tick stage, tick age, and seasonal treatment, the number of infected mice, the number of ticks per mouse, and the total number of ticks are shown*.

### Seasonal Treatment of the 4-Month-Old Nymphs

When the 4-month-old nymphs were 1 month old, they were randomly assigned to one of three seasonal treatments: artificial summer, artificial winter, and natural winter (Figure 1C), hereafter referred to as phytotron, fridge, and underground, respectively. These three treatments were chosen to simulate summer conditions (warm temperature and long photoperiod) and winter conditions (cold temperature and no light) that are experienced by *I. ricinus* ticks in the natural environment. We chose to have no light in both winter treatments to simulate the natural situation. *Ixodes* ticks encounter very little light during the winter because they hide in the soil and under the leaf litter to protect themselves from cold temperatures (Dusbabek et al., 1971; Daniel et al., 1972; Gray et al., 2016). The nymphs spent a total of 3 months in these three seasonal treatments (22 January 2016–15 April 2016).

In the artificial summer treatment, the nymphs were kept in a phytotron [4–5 h: 1 lumen (lm), 21.5°C; 5–19 h: 2 lm, 25°C; 19–20 h: 1 lm, 21.5°C; 20–4 h; 0 lm, 18°C] with a relative humidity of 85%. In the artificial winter treatment, the nymphs were kept in a fridge at a temperature of 4°C and with no light. For the natural winter treatment, the nymphs were kept in a plastic box (30 cm × 23 cm × 10 cm) that was buried in the soil at a depth of 10 cm in a forest in the botanical garden of Neuchâtel. The box contained three button logs that measured the temperature every 30 min. The mean daily average temperature in the natural winter treatment was 6.44°C (range = 3.72–10.50°C; for details see Section 1 in the Supplementary Material). In each of the three seasonal treatments, the nymphs were kept in their individual Eppendorf tubes.

Four months after the larva-to-nymph molt (15 April 2016), the 4-month-old nymphs were checked to determine whether they were alive or dead. The survival was very high, only 4 of the 444 nymphs had died (3, 0, and 1 in the artificial summer, artificial winter, and natural winter, respectively). The next day (16 April 2016), all live 4-month-old nymphs were frozen at −20°C. The seasonal treatments, mouse sample sizes, and tick sample sizes are shown in Table 1.

### DNA Extraction

The engorged larvae, 1-month-old nymphs, and 4-month-old nymphs were crushed in a TissueLyser II (Figure 1D) using a previously described protocol (Jacquet et al., 2015; Genné et al., 2018). The crushed ticks were digested with proteinase K at 56°C overnight. Tick DNA was extracted using QIAGEN DNeasy 96 Blood and Tissue kit well-plates and following the QIAGEN protocol. Each plate contained two *Anopheles gambiae* mosquitoes as negative *B. afzelii* DNA extraction controls. For each sample, the DNA was eluted into 65 μl of water.

### General and Strain-Specific qPCR Assays

The *B. afzelii* infection status of the ticks was assessed with a qPCR assay that targets a 132-bp fragment of the *flagellin* gene (Figure 1D), as described previously (Genné et al., 2018). The identities of the strains in the nymphs were determined using two strain-specific qPCRs (Figure 1D). These qPCR assays amplify the same 143-bp fragment of the *ospC* gene but use different probes that are specific for *ospC* alleles A3 and A10, as described previously (Genné et al., 2018). For each qPCR reaction, 3 μl of DNA template was used. The qPCRs were done using a LightCycler® 96 Multiwell Plate white (Roche). All the plates contained 80 tick DNA samples, 2 negative *B. afzelii* DNA extraction controls (mosquitoes), 2 negative controls for the qPCR (PCR-grade water), and 12 positive controls. For each of the three different qPCR assays (*flagellin, ospC* A3, and *ospC* A10), a sample of 81 ticks was tested twice to determine the repeatability of the assay (Genné et al., 2018). For the *flagellin, ospC* A3, and *ospC* A10 qPCR, the repeatability of the log10-transformed spirochete loads was 98.3%, 97.8%, and 97.0%, respectively (Genné et al., 2018).

### Statistical Analyses

#### *B. afzelii* Infection Status of Immature *I. ricinus* Ticks

The *flagellin* qPCR and the *ospC* qPCRs were used to determine the *B. afzelii* infection status of the ticks. Samples were excluded from the analysis whenever the *ospC* qPCR and the *flagellin* qPCR differed with respect to the infection status of the tick. Generalized linear mixed effects models (GLMMs) with binomial errors were used to analyse the infection status of the ticks (0 = uninfected, 1 = infected). The fixed factor that combined tick age class and seasonal treatment had 5 levels: engorged larvae, 1-month-old nymphs, phytotron 4-month-old nymphs, fridge 4-month-old nymphs, and underground 4-month-old nymphs. These levels were combined to create new factors with fewer levels: tick age (3 levels: larvae, 1-month-old nymphs, and 4-month-old nymphs), and tick stage (2 levels: larvae and nymphs). Mouse ID was modeled as a random factor. To determine the statistical significance of the fixed factors, models that differed with respect to the fixed factor of interest were compared using log-likelihood ratio (LLR) tests. Similarly, comparing a model that includes the 5 levels of tick age class and seasonal treatment with a model that contains 3 levels of tick age, tests whether the seasonal treatment had a significant effect on the phenotype of the 4-month-old nymphs.

#### *B. afzelii* Spirochete in Immature *I. ricinus* Ticks

The total spirochete abundance inside the ticks (hereafter referred to as total spirochete load) was analyzed for the subset of ticks infected with *B. afzelii*. The total estimate of the spirochete load inside each tick was estimated by correcting the spirochete load in 3 μl of DNA template to the total DNA extraction elution volume (i.e., multiplied by a factor of 21.67 = 65 μl/3 μl). The *flagellin* qPCR was used to estimate the total spirochete load in the ticks. In co-infected ticks, the strain-specific spirochete loads estimated by the *ospC* qPCRs were constrained to sum to the total spirochete load estimated by the *flagellin* qPCR (Genné et al., 2018). Linear mixed effects models (LMMs) were used to analyse the spirochete loads, which were log_10_-transformed to normalize the residuals. The fixed factors were the same as for the GLMM of infection status. Mouse ID was modeled as a random factor.

#### Competition Between Strains Over the Life Cycle of the Immature Tick

GLMMs with binomial errors and LMMs with normal errors were used to analyse the strain-specific prevalence and the strain-specific spirochete load, respectively. The fixed factor that combined tick age class and seasonal treatment (5 levels: engorged larvae, 1-month-old nymphs, phytotron 4-month-old nymphs, fridge 4-month-old nymphs, and underground 4-month-old nymphs), the strain (2 levels: Fin-Jyv-A3, NE4049), competition (2 levels: no, yes), and their interactions were fixed factors. Mouse identity was included as a random factor. As before, we tested whether the 5-level factor of tick age class and seasonal treatment could be reduced to tick age (3 levels: larvae, 1-month-old nymphs, and 4-month-old nymphs), or tick stage (2 levels: larvae and nymphs). To determine the statistical significance of the fixed factors, models that differed with respect to the fixed factor of interest were compared using LLR tests. The results from this stepwise model simplification approach were compared with a model selection approach based on the Akaike information criterion (AIC).

#### Statistical Software

The statistical analyses were done in R v. 1.1.463 and using the following R packages: base, lme4, emmeans, and MuMIn. The “lmer” and “glmer” functions (lme4 package) were used to create the GLMMs and LMMs. The “anova” function (base package) was used to perform the log-likelihood ratio tests. The “model.sel” function (MuMIn package) was used to perform the AIC-based model selection. The “emmeans,” “contrast,” and “pairs” functions (emmeans package) were used for the *post-hoc* analyses of the GLMMs and LMMs (details of the *post-hoc* tests are shown in Section 8 of the Supplementary Material).

## Results

### Mice

Of the 40 mice used in this study, 2 mice died (S5 and S12), 1 mouse did not become infected (S37), and 4 mice in the co-infected treatments acquired only one strain (S17, S20, S25, S29). Ticks produced by these 7 mice were excluded from the study. For the engorged larvae and the 1-month-old nymphs, the final sample size was 33 infected mice that were distributed across the four infection treatments as follows: Fin-Jyv-A3 (*n* = 9), Fin-Jyv-A3 + NE4049 (*n* = 7), NE4049 (*n* = 9), and NE4049 + Fin-Jyv-A3 (*n* = 8). For the 4-month-old nymphs, the final sample size was 26 infected mice because some mice did not produce enough ticks to be included in this tick age group (Table 1).

### Ticks

Of the 849 ticks in this study, 49 were excluded because the *flagellin* qPCR and the *ospC* qPCR disagreed with respect to tick infection status. Specifically, this criterion excluded 13.9% (23/165) of the larvae, 5.3% (17/318) of the 1-month-old nymphs, and 2.5% (9/366) of the 4-month-old nymphs. The final sample sizes for each of the five groups were as follows: engorged larvae (*n* = 142), 1-month-old nymphs (*n* = 301), 4-month-old phytotron nymphs (*n* = 119), 4-month-old fridge nymphs (*n* = 120), and 4-month-old underground nymphs (*n* = 118).

### Prevalence of *B. afzelii* in Immature Ticks

The infection prevalence is defined as the percentage of ticks that tested positive for *B. afzelii* (ignoring strain identity). The infection prevalence for the different tick ages (Table 2) was as follows: larvae (37.3% = 53/142), 1-month-old-nymphs (87.7% = 264/301), and 4-month-old nymphs (82.6% = 295/357). After combining all the nymphs, the prevalence of *B. afzelii* infection in the nymphs (85.0% = 559/658) was 2.3 times higher compared to the larvae (37.3% = 53/142). The statistical analysis of these infection prevalences is presented in the next section.

**Table 2:** The prevalence of *B. afzelii* infection in *I. ricinus* ticks and the *B. afzelii* spirochete loads in the subset of infected *I. ricinus* ticks are shown separately for the seasonal treatment, tick age, and tick stage.

**Factor**	**Level**	**Inf/Total**	**Prev (%)**	**Mean**	**95% CI**
Treatment	Larva	53/142	37.3%	429	286–644
Treatment	1-month-old nymph	264/301	82.6%	5,055	4,215–6,063
Treatment	4-month-old phytotron nymph	95/119	79.8%	2,651	1,959–3,589
Treatment	4-month-old fridge nymph	98/120	81.7%	2,928	2,173–3,945
Treatment	4-month-old underground nymph	102/118	86.4%	3,477	2,596–4,657
Tick age	Larva	53/142	37.3%	429	286–644
Tick age	1-month-old nymph	264/301	87.7%	5,055	4,216–6,062
Tick age	4-month-old nymph	295/357	82.6%	3,009	2,534–3,574
Tick stage	Larva	53/142	37.3%	429	285–647
Tick stage	Nymph	559/658	85.0%	3,845	3,388–4,363

*The spirochete load is the total number of spirochetes in the tick and is based on the flagellin qPCR. Shown are the geometric mean of the spirochete loads and the 95% confidence intervals (95% CI)*.

### Effect of Tick Age and Tick Seasonal Treatment on the Prevalence of *B. afzelii* Infection

We analyzed the prevalence of *B. afzelii* infection in the immature *I. ricinus* ticks (ignoring strain identity) as a function of tick age and seasonal treatment. The seasonal treatment had no effect on the infection prevalence and we therefore combined all the 4-month-old nymphs into a single group (see Section 2 in the Supplementary Material).

There was a significant effect of age on infection prevalence (LLR test of age vs. null: Δ df = 2, Δ dev = 146.16 *p* < 0.0001). The infection prevalence in the larvae was significantly lower compared to the 1-month-old nymphs (emmeans: *p* < 0.0001) and compared to the 4-month-old nymphs (emmeans: *p* < 0.0001). The infection prevalence was not significantly different between the 1-month-old nymphs and the 4-month-old nymphs (emmeans: *p* = 0.249).

### Spirochete Load of *B. afzelii* in Immature Ticks

The spirochete load is defined as the total number of spirochetes in an infected tick (ignoring strain identity). The geometric mean spirochete loads were calculated for the subset of infected ticks (Table 2) and were as follows: larvae (*n* = 53; mean = 429; range = 28–269,154), 1-month-old nymphs (*n* = 264; mean = 5,055; range = 34–218,776), and 4-month-old nymphs (*n* = 295; mean = 3,009; range = 36–275,423). The spirochete load in the 1-month-old nymphs was 11.8 times higher compared to the larvae and 1.7 times higher compared to the 4-month-old nymphs. Over the 3-month overwintering period, the nymphs lost spirochetes at a rate of 22.7 spirochetes per day. The statistical analysis of these spirochete loads is presented in the next section.

### Effect of Tick Age and Tick Seasonal Treatment on the *B. afzelii* Spirochete Load

We analyzed the total *B. afzelii* spirochete load in the immature *I. ricinus* ticks (ignoring strain identity) as a function of tick age and seasonal treatment. The seasonal treatment had no effect on the nymphal spirochete load and we therefore combined all the 4-month-old nymphs into a single group (see Section 3 in the Supplementary Material).

**Figure 2:**
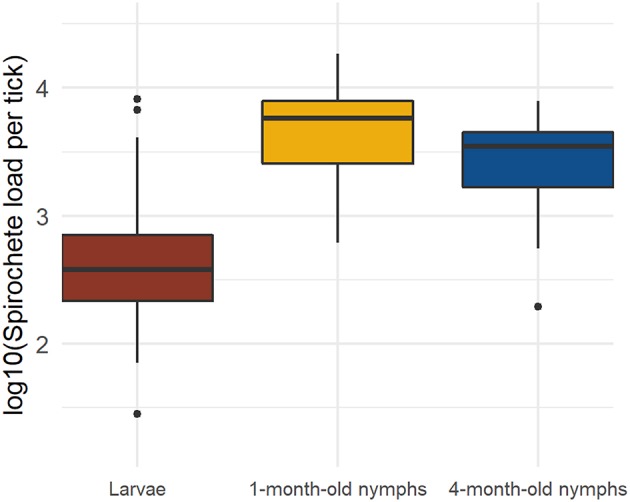
The *B. afzelii* spirochete loads in immature *I. ricinus* ticks change with the age of the tick. The spirochete load is lowest in the engorged larvae, highest in 1-month-old nymphs, and intermediate in 4-month-old nymphs. Each data point represents the mean for ticks sampled from the same mouse. The total numbers of ticks for each of the three tick ages are as follows: engorged larvae (*n* = 142), 1-month-old nymphs (*n* = 301), and 4-month-old nymphs (*n* = 357). The boxplot shows the medians (black line), the 25th and 75th percentiles (edges of the box), the minimum and maximum values (whiskers), and the outliers (solid circles).

There was a significant effect of tick age on the tick spirochete load (LLR test of age vs. null: Δ df = 2, Δ dev = 124.65, *p* < 0.0001; Table 2, Figure 2). The spirochete loads in the engorged larvae were significantly lower compared to the 1-month-old nymphs (emmeans: *p* < 0.0001) and compared to the 4-month-old nymphs (emmeans: *p* < 0.0001). The 1-month-old nymphs had a significantly higher spirochete load than the 4-month-old nymphs (emmeans: *p* = 0.0002). In summary, the spirochete load was lowest in the larvae, highest in the 1-month-old nymphs, and intermediate in the 4-month-old nymphs (Table 2, Figure 2).

### Effect of Competition Between Strains on the Strain-Specific Transmission to Immature *I. ricinus* Ticks

Here the response variable is the strain-specific infection prevalence in the immature ticks, as estimated by the *ospC* qPCR. To be conservative, the competition factor (two levels: no, yes) was based on the co-infection status of the mice rather than the co-infection status of the ticks (see Section 4 in the Supplementary Material). Model comparison found justification for combining all of the nymphs into a single group (see Section 5 in the Supplementary Material). Thus, the strain-specific prevalence was analyzed as a function of three fixed factors: strain, competition, tick stage, and their interactions.

A classic step-wise model simplification approach using LLR tests found that the best model included strain, competition, tick stage, the competition: tick stage interaction, and the strain:competition interaction (see Section 5 in the Supplementary Material). The AIC-based model selection approach converged on the same model (see Section 7 in the Supplementary Material). The presence of two significant two-way interactions complicates the interpretation of the main effects and requires splitting the statistical analyses. We ran separate analyses for larvae and nymphs; this approach allowed us to independently test the effects of competition and strain for each tick stage.

**Figure 3:**
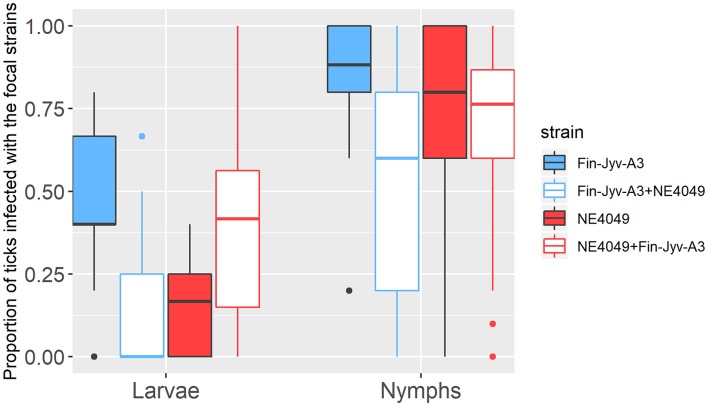
The proportion of immature *I. ricinus* ticks infected with *B. afzelii* is shown as a function of three factors: (1) tick age (engorged larvae, 1-month-old nymphs, and 4-month-old nymphs), (2) strain (Fin-Jyv-A3 in blue and NE4049 in red), and (3) competition (competition absent in solid colors vs. competition present in white). The proportion of ticks infected with the focal strain is an estimate of strain-specific host-to-tick transmission. The graph shows the raw data and not all observable differences are statistically significant. According to the parameter estimates of our statistical analysis, competition between strains in co-infected mice reduced host-to-tick transmission of strain Fin-Jyv-A3 to engorged larvae and nymphs and reduced the host-to-tick transmission of strain NE4049 to nymphs. In contrast, competition between strains in co-infected mice did not influence the host-to-tick transmission of strain NE4049 to engorged larvae. Each data point represents the mean for a single mouse. The boxplots show the medians (black line), the 25th and 75th percentiles (edges of the box), the minimum and maximum values (whiskers), and the outliers (solid circles).

In the larvae, the interaction between strain and competition was significant (GLME LLR: Δ df = 1, Δ dev = 8.228, *p* = 0.004), the effect of competition was therefore tested separately for each strain. For strain Fin-Jyv-A3, its prevalence was higher in larvae that fed on singly infected mice (45.5% = 20/44) compared to larvae that fed on co-infected (14.8% = 4/27) mice, and this difference was significant (GLME LLR: Δ df = 1, Δ dev = 5.173, *p* = 0.023). For strain NE4049, its prevalence was lower in larvae that fed on singly infected mice (15.0% = 6/40) compared to larvae that fed on co-infected mice (35.5% = 11/31), but this difference was not significant (GLME LLR: Δ df = 1, Δ dev = 3.16, *p* = 0.075). In summary, competition between strains significantly reduced transmission to engorged larval ticks for strain Fin-Jyv-A3 but not strain NE4049 (Table 3, Figure 3, Figure S3).

**Table 3:** The strain-specific infection prevalences in the immature *I. ricinus* ticks are shown for each of the eight unique combinations of tick stage, *B. afzelii* strain, and competition.

**Stage**	**Strain**	**Competition**	**% Infected**
Larvae	Fin-Jyv-A3	No	20/44 (45.5%)
Larvae	Fin-Jyv-A3	Yes	4/27 (15.8%)
Larvae	NE4049	No	6/40 (15.0%)
Larvae	NE4049	Yes	11/31 (35.5%)
Nymphs	Fin-Jyv-A3	No	127/147 (86.4%)
Nymphs	Fin-Jyv-A3	Yes	79/157 (50.3%)
Nymphs	NE4049	No	152/194 (78.4%)
Nymphs	NE4049	Yes	109/160 (68.1%)

In the nymphs, the interaction between strain and competition was not significant (GLME LLR: Δ df = 1, Δ dev = 2.724, *p* = 0.091) and was removed from the model. Competition was significant (GLME LLR: Δ df = 1, Δ dev = 8.683, *p* = 0.003), but strain was not (GLME LLR: Δ df = 1, Δ dev = 0.024, *p* = 0.877). The prevalence of both strains was higher in nymphs that had fed as larvae on singly infected mice (81.8% = 279/341) compared to nymphs that had fed as larvae on co-infected mice (59.3% = 188/317; Table 3, Figure 3, Figure S3).

### Effect of Competition Between Strains on the Strain-Specific Spirochete Load in Immature *I. ricinus* Ticks

Here the response variable is the strain-specific spirochete load in the immature ticks, as estimated by combining the *ospC* qPCR and the *flagellin* qPCR. Model comparison showed that we could combine all of the 4-month-old nymphs into a single group (see Section 6 in the Supplementary Material). Thus, the strain-specific spirochete load was analyzed as a function of three fixed factors: strain, competition, tick age and their interactions.

A classic stepwise model simplification approach using LLR tests found that none of the interactions were significant and that the best model contained the three fixed factors of strain, competition, and tick age (see Section 6 in the Supplementary Material). The AIC-based model selection approach converged on the same model (see Section 7 in the Supplementary Material).

**Figure 4:**
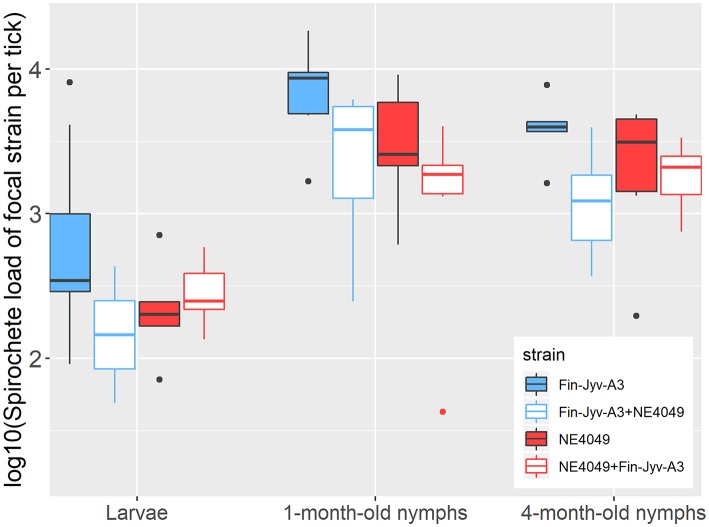
The *B. afzelii* spirochete loads in immature *I. ricinus* ticks are shown as a function of three factors: (1) tick age (engorged larvae, 1-month-old nymphs, and 4-month-old nymphs), (2) strain (Fin-Jyv-A3 in blue and NE4049 in red), and (3) competition (competition absent in solid colors vs. competition present in white). The strain-specific spirochete loads were log10-transformed to normalize the residuals. The graph shows the raw data and not all observable differences are statistically significant. According to the parameter estimates of our statistical analysis, competition between strains reduced the spirochete loads of both strains in all three age classes of ticks. Our statistical analysis also found that strain Fin-Jyv-A3 had a higher spirochete load in immature *I. ricinus* ticks compared to strain NE4049. Each data point represents the mean for a single mouse. The boxplots show the medians (black line), the 25th and 75th percentiles (edges of the box), the minimum and maximum values (whiskers), and the outliers (solid circles).

The three fixed factors all had significant effects on the spirochete load: tick age (LLR test: Δ df = 2, Δ dev = 86.393, *p* = < 0.001), strain (LLR test: Δ df = 1, Δ dev = 3.948, *p* = 0.047), and competition (LLR test: Δ df = 1, Δ dev = 10.589, *p* = 0.011) (Figure 4, Figure S4). The spirochete load in the engorged larvae was significantly lower compared to the 1-month-old nymphs (emmeans: *p* < 0.0001) and the 4-month-old nymphs (emmeans: *p* < 0.0001) (Table 3, Figure 4, Figure S4A). The 1-month-old nymphs had a higher spirochete load than the 4-month-old nymphs (emmeans: *p* < 0.0046; Table 3, Figure 4, Figure S4A). According to our parameter estimates, strain Fin-Jyv-A3 always had a higher spirochete load in the tick than strain NE4049 (raw data in Figure 4, parameter estimates in Figure S4B, Table S3). Competition reduced the spirochete load of each strain and this effect was the same for engorged larvae, 1-month-old nymphs, and 4-month-old nymphs (raw data in Figure 4, parameter estimates in Figure S4C). In all three of these tick ages, the spirochete load of each strain was reduced in ticks that had fed on co-infected mice compared to ticks that had fed on mice infected with single strains (raw data in Figure 4, Table 4, parameter estimates in Figure S4A, Table S3).

**Table 4:** The strain-specific spirochete loads in the subset of infected *I. ricinus* ticks are shown for each of the 12 unique combinations of tick age, *B. afzelii* strain, and competition.

**Tick age**	**Strain**	**Competition**	**Mean**	**95% CI**
Larva	Fin-Jyv-A3	No	513	241–1,090
Larva	Fin-Jyv-A3	Yes	146	27–786
Larva	NE4049	No	209	53–829
Larva	NE4049	Yes	338	122–934
1-month-old nymph	Fin-Jyv-A3	No	7,111	4,873–10,376
1-month-old nymph	Fin-Jyv-A3	Yes	3,352	1,972–5,697
1-month-old nymph	NE4049	No	3,493	2,394–5,098
1-month-old nymph	NE4049	Yes	1,875	1,216–2,892
4-month-old nymph	Fin-Jyv-A3	No	3,951	2,738–5,703
4-month-old nymph	Fin-Jyv-A3	Yes	1,410	923–2,154
4-month-old nymph	NE4049	No	2,520	1,851–3,429
4-month-old nymph	NE4049	Yes	1,960	1,350–2,847

*Mean is the geometric mean of the strain-specific spirochete loads on the original scale. The 95% confidence interval (95% CI) for the geometric mean is also shown*.

## Discussion

### Competition in the Rodent Host Reduces Host-to-Tick Transmission

Our study found that co-infection in the rodent host reduced host-to-tick transmission success of both strains of *B. afzelii* to *I. ricinus* nymphs. This result is in agreement with other studies that have investigated how mixed strain infections of *B. burgdorferi* sl in the rodent host influence strain-specific transmission to *Ixodes* ticks (Derdáková et al., 2004; Rynkiewicz et al., 2017; Genné et al., 2018). Studies on other vector-borne pathogens have also shown that competition between strains in the vertebrate host reduces strain-specific transmission to the arthropod vector (de Roode et al., 2005b; Reif et al., 2014), suggesting that this result is a general phenomenon in these systems. To date, all *B. burgdorferi* sl studies on the relationship between co-infection and host-to-tick transmission have measured the latter phenotype in young nymphs (i.e., sacrificed shortly after the larva-to-nymph molt) that have not overwintered (Derdáková et al., 2004; Rynkiewicz et al., 2017; Genné et al., 2018). In nature, the majority of *Ixodes* nymphs overwinter and search for a host the following spring when they are much older (Gray, 1984, 1991; Gray et al., 2016). Hence, a new contribution of our study was to show that the effects of inter-strain competition in the rodent host persist in *I. ricinus* nymphs that were allowed to overwinter and age. We found that co-infection reduced the host-to-nymph transmission of both strains, which is different from previous studies (including ours) that found asymmetric competition (Rynkiewicz et al., 2017; Genné et al., 2018).

Interestingly, our study shows that reduced host-to-tick transmission can be detected in engorged larval ticks immediately following drop-off. This observation reinforces the idea that competition between strains in the tissues of the rodent host (Strandh and Råberg, 2015) reduces the probability that a given strain will colonize a feeding larval tick. The much lower infection prevalence in the engorged larvae compared to the nymphs is due to their much lower spirochete load, which the qPCR then fails to detect. However, an alternative explanation for the observation that the infection prevalence and spirochete load in engorged larvae are lower compared to the nymphs is that mouse blood interferes with the efficacy of the qPCR assays (Schrader et al., 2012; Buckwalter et al., 2014; Sidstedt et al., 2018). We consider this explanation unlikely because an early study that used immunofluorescence microscopy also showed that *Ixodes scapularis* larvae acquire a small inoculum of *B. burgdorferi* sensu stricto (ss) spirochetes, which subsequently expands during the period of larva-to-nymph development (Piesman et al., 1990). Similarly, a recent study on the population dynamics of *B. afzelii* in immature *I. ricinus* ticks showed that qPCR and immunofluorescence microscopy gave the same results (Pospisilova et al., 2019). In summary, there is a general consensus that *Ixodes* larvae acquire a small inoculum of *B. burgdorferi* sl spirochetes, which grows to a larger size in the tick midgut during the period of larva-to-nymph development (Piesman et al., 1990; Soares et al., 2006; Jacquet et al., 2017; Pospisilova et al., 2019).

### Competition in the Arthropod Vector

To date, very few studies have investigated interactions between pathogen strains in the arthropod vector (Reif et al., 2014; Pollitt et al., 2015; Genné et al., 2018). Inter-strain facilitation has been shown for malaria parasites in Anopheline mosquitoes (Pollitt et al., 2015), whereas inter-strain competition has been shown for *Francisella novicida* in *Dermacentor andersoni* ticks (Reif et al., 2014), and for *Borrelia afzelii* in *Ixodes ricinus* ticks (Genné et al., 2018). Our study found that competition resulted in decreased spirochete loads for both strains and in all three tick age classes in this study: engorged larvae, 1-month-old nymphs, and 4-month-old nymphs. This result is in agreement with our previous study, which was restricted to the 1-month-old nymphs (Genné et al., 2018). The present study is an improvement on our previous study because it is based on a larger sample size (800 ticks vs. 301 ticks) and because the results are more general; i.e., we show here that inter-strain competition occurs at three different time points over the first 5 months of the life cycle of immature *I. ricinus* ticks compared to only one time point (Genné et al., 2018). An important remaining question is whether competition between strains of *B. burgdorferi* sl in the nymph influences subsequent nymph-to-host transmission of the bacterium.

### Dynamics of *B. burgdorferi* sl Spirochete Populations in Immature *Ixodes* Ticks

The population size of *B. burgdorferi* sl spirochetes is highly dynamic over the life cycle of immature *Ixodes* ticks (Piesman et al., 1990, 2001; De Silva and Fikrig, 1995; Soares et al., 2006; Jacquet et al., 2017; Pospisilova et al., 2019). A recent study on the spirochete population dynamics of *B. afzelii* in *I. ricinus* found that engorged larvae acquired a small inoculum (~600 spirochetes), which subsequently expanded 34-fold to reach a peak spirochete population size (21,005 spirochetes) in 2-week-old nymphs (Pospisilova et al., 2019). The results of our study were very similar; the engorged larvae acquired a small inoculum of *B. afzelii* (~400 spirochetes), which expanded and was 12 times higher in the 1-month-old nymphs (5,005 spirochetes). Numerous studies on *B. burgdorferi* ss in *I. scapularis* have likewise shown that the spirochete population size increases dramatically after a blood meal in both larvae and nymphs (Piesman et al., 1990, 2001; De Silva and Fikrig, 1995; Soares et al., 2006).

The ability of *B. burgdorferi* sl to persist inside the nymph over long periods of time (8–12 months) is critical for the maintenance of Lyme disease in nature (Fazzino et al., 2015). A recent study on the spirochete population dynamics of *B. afzelii* in *I. ricinus* nymphs found that the spirochete population size decreased by 71.3% between 2 and 6 weeks post-molt but reached a stable plateau after this period (Pospisilova et al., 2019). We have observed a similar decrease in spirochete population size between 1-month-old and 4-month-old nymphs in the present study (decrease of 40.5%), and in a previous study (85.8% decrease) (Jacquet et al., 2017). As we only measured the nymphal spirochete population size at two time points, we do not know whether it plateaued, as was shown in the other study (Pospisilova et al., 2019). A recent study on the genetic mechanisms underlying persistence of *B. burgdorferi* ss in *I. scapularis* found no evidence that the spirochete population size declined over time (Fazzino et al., 2015).

One explanation for the observed decrease in the spirochete population over time is bloodmeal digestion, which is a slow process in ixodid ticks that occurs in the gut epithelium rather than the gut lumen (Sonenshine, 1991; Horn et al., 2009; Sojka et al., 2013). Proteomics studies have shown that digestion of the larval blood meal causes host proteins to decrease slowly over time, and some can be identified 10 months after the larva-to-nymph molt (Wickramasekara et al., 2008; Laskay et al., 2013). The slow digestion of the blood meal could explain the gradual decrease of the spirochetes over time in the tick midgut (Kung et al., 2013). We expected that the intensity of inter-strain competition would increase with this age-related decline in the spirochete population, but we found no evidence that competition was stronger in the 4-month-old nymphs compared to the 1-month-old nymphs. One limitation of our qPCR-based approach is that it allows us to estimate spirochete abundance but not spirochete viability, which may decline with advanced nymphal age.

### Mechanisms of Competition Inside the Tick Vector

The mechanisms underlying the competition between *B. afzelii* strains in the tick remain unknown. Competition between parasite strains is classified into three different types: interference, exploitation, and apparent competition (Mideo, 2009). Interference competition is unlikely in *B. burgdorferi* sl, because spirochetes are not known to produce toxic substances (Tilly et al., 2008). In exploitation competition, strains compete over limited resources like space or nutrients. This type of competition is expected because nutrients in the tick midgut will disappear over time as the tick digests its bloodmeal (Laskay et al., 2013). Furthermore, studies have shown that ticks contain a relatively small number of spirochetes, suggesting that strains compete over space as well (Durand et al., 2017; Genné et al., 2018). In apparent competition, interactions between strains are mediated by the host immune system. The innate immune system of ticks uses antimicrobial peptides (e.g., lysozymes and defensins) as defense against microbial pathogens (Kopáček et al., 2010; Hajdušek et al., 2013). Spirochetes in the tick hemolymph are cleared by hemocytes using phagocytosis (Rittig et al., 1996; Coleman et al., 1997; Johns et al., 2001). However, we are not aware of any experimental evidence demonstrating that the tick immune system mediates competition between strains of *B. burgdorferi* sl.

### Inter-strain Competition and Nymph-to-Host Transmission

Our study found that competition between strains reduced the strain-specific spirochete loads in *I. ricinus* nymphs. An important question is whether this inter-strain competition influences the subsequent nymph-to-host transmission success of the strains. Most *B. afzelii* spirochetes spend 8 months or more in the nymph midgut before achieving nymph-to-host transmission (Gray et al., 2016). We expect that the temporal decline in quantity or viability of the nymphal spirochete population could eventually result in the loss of the less abundant strains from the co-infected nymph (i.e., competitive exclusion). Studies on *B. burgdorferi* ss and *F. novicida* have found that ticks can limit the number of strains that are transmitted to the vertebrate host (Rego et al., 2014; Reif et al., 2014). The biology of spirochetes in blood-feeding nymphs further suggests that inter-strain competition could reduce strain-specific nymph-to-host transmission. During the nymphal blood meal, spirochetes migrate from the midgut to the salivary glands (Spielman et al., 1987; Zung et al., 1989), where their abundance is surprisingly low (Piesman et al., 2001). One study suggested that nymphs inoculate only ~100 spirochetes into the vertebrate host (Kern et al., 2011). These observations suggest that *B. burgdorferi* sl strains that are the most abundant in co-infected nymphs are more likely to be transmitted to the reservoir host during the nymphal blood meal (Durand et al., 2017). Future experimental infection studies should test whether co-infection in the nymph reduces strain-specific nymph-to-host transmission.

### Relevance of Our Study to the Situation in Nature

The results of our laboratory experiment are relevant to understanding what happens in nature. Studies on wild *I. ricinus* nymphs found that 77–79% of the nymphs are co-infected with multiple strains of *B. afzelii* and that nymphs carry an average of 2.4–2.9 strains (Durand et al., 2015, 2017). Numerous studies in North America have found that wild *I. scapularis* ticks are commonly infected with multiple strains of *B. burgdorferi* ss (Wang et al., 1999; Qiu et al., 2002; Brisson and Dykhuizen, 2004; Walter et al., 2016; Di et al., 2018). Thus, co-infected nymphs are the norm rather than the exception in areas where Lyme disease is endemic. A field study on *B. afzelii* in wild *I. ricinus* nymphs found indirect evidence that competition between strains in nymphs reduces the strain-specific abundance and strain-specific nymph-to-host transmission (Durand et al., 2017). First, this study found the same pattern that we found in the present study: co-infection in the nymph reduced the abundance of the constituent strains (Durand et al., 2017). Second, this study found a positive correlation between the strain-specific spirochete load and the strain-specific prevalence suggesting that *B. afzelii* strains with higher nymphal abundance have higher nymph-to-host transmission (Durand et al., 2017). In summary, our controlled experiments found patterns of co-infection and competition that we have also found in surveys of wild *I. ricinus* populations.

### No Effect of Seasonal Treatment on Spirochete Load

We found no effect of seasonal treatment on the *B. afzelii* spirochete load in the 4-month-old nymphs. The seasonal treatments were chosen to simulate summer conditions (mean temperature of 22.4°C and 16 h light: 8 h darkness) and winter conditions (mean temperature of 4 and 6.44°C and 24 h darkness) that are experienced by *I. ricinus* ticks in the natural environment (Dusbabek et al., 1971; Daniel et al., 1972; Gray et al., 2016). We expected that the nymphs exposed to summer conditions would have higher metabolism, faster digestion, and therefore lower spirochete population sizes in their midguts than nymphs exposed to winter conditions. We were surprised that the seasonal treatment had no effect on the spirochete population size in the nymphal midgut. This result suggests that the large differences in temperature and light conditions between the summer and winter conditions over a period of 3 months was not enough to influence tick metabolism, tick digestion of spirochetes, and the dynamics of the *B. afzelii* spirochete population inside the nymphs. Future studies should investigate whether more extreme conditions (e.g., hotter and colder temperatures) would influence the spirochete population dynamics and inter-strain competition inside the nymph vector. We had previously suggested that the temporal decline in nymphal spirochete load following the larva-to-nymph molt could be an artifact of housing our ticks under unnatural “summer-like” conditions in the lab (Jacquet et al., 2017). Thus, the present study demonstrates that the nymphal spirochete load also decreases over time when nymphs are housed under more natural “winter-like” conditions. The survival of the nymphs over the 3-month winter treatments was very high (99.1%) due to the mild temperatures (6.44°C in natural winter and 4.00°C in artificial winter). Storing ticks in separate Eppendorf tubes and burying them underground is a promising approach for measuring individual tick survival under natural winter conditions.

### The Infectious Dose and the Importance of Using Ticks Instead of Needles

In studies that investigate competition between pathogen strains, it is critical to control the infectious dose (ID) with which the vertebrate host is infected (de Roode et al., 2005b). If the ID was consistently larger for one strain, we would expect this strain to have higher competitive success (all else being equal). There is evidence that when mice are infected with *B. burgdorferi* ss using artificial needle inoculation, a 10-fold difference in the ID can influence the spirochete load in the mouse tissues (Ma et al., 1998), and presumably the efficiency of host-to-tick transmission. In the Methods, we assumed that the ID delivered by the nymphs is proportional to the product of the number of infected nymphs and the mean spirochete load per infected nymph, and we estimated that the ID for strain Fin-Jyv-A3 was two times higher than strain NE4049. Despite having a (theoretical) 2-fold disadvantage in abundance during the nymphal tick bite (Figure 1A), strain NE4049 was still able to reduce the performance of strain Fin-Jyv-A3. This observation suggests that competitive interactions would remain even if both strains had the exact same ID.

Alternatively, one could infect mice via needle inoculation of spirochete cultures, which can be more easily quantified, but this artificial approach creates other problems. Tick saliva enhances the ability of *B. burgdorferi* sl pathogens to infect their vertebrate host (Kazimírová and Stibraniova, 2013). For example, cultured spirochetes are an order of magnitude less infectious than the spirochetes in tick salivary glands (Xu et al., 1996; Lima et al., 2005). Tick saliva also increases the spirochete load of *B. burgdorferi* sl in rodent tissues (Zeidner et al., 2002). A recent study found that the mode of inoculation (needle vs. tick) influenced tissue tropism of *B. burgdorferi* sl (Sertour et al., 2018). In summary, there is lots of evidence that the mode of inoculation influences the infection phenotype. *B. burgdorferi* sl spirochetes have co-evolved with ticks and not needles. Using ticks will generally give a better reflection of what occurs in nature.

## Conclusion

This study found that co-infection in the mice reduced the strain-specific host-to-tick transmission success to larval ticks and that this effect was maintained for both strains in the resultant nymphs at 1 and 4 months after the larva-to-nymph molt. This study also demonstrates that competition between strains of *B. afzelii* occurs in co-infected immature *I. ricinus* ticks. Inter-strain competition resulted in decreased spirochete loads for both strains in all three ages of immature ticks investigated in our study. The 3-month-long seasonal treatments did not affect the strain-specific spirochete load nor the intensity of inter-strain competition in the 4-month-old nymphs. Future studies should investigate whether inter-strain competition in the nymph influences the strain-specific nymph-to-host transmission success.

## Data Availability Statement

The raw data for this study are stored on Zenodo (https://doi.org/10.5281/zenodo.3569759) in an Excel file titled “Raw data.xlsx.”

## Ethics Statement

The commission that is part of the “Service de la Consommation et des Affaires Vétérinaires (SCAV)” of Canton Vaud, Switzerland evaluated and approved the ethics of this study. The Veterinary Service of the Canton of Neuchâtel, Switzerland issued the animal experimentation permit used in this study (NE04/2016).

## Author Contributions

DG and MV conceived and designed the study, and wrote the manuscript. DG and AS conducted the experiment and performed the molecular work. OR helped with the experimental infections. DG conducted the statistical analyses. All authors read and approved the final version of the manuscript.

### Conflict of Interest

The authors declare that the research was conducted in the absence of any commercial or financial relationships that could be construed as a potential conflict of interest.
