# Supplementary material for: Competition Between Strains of Borrelia afzelii in Immature Ixodes ricinus Ticks Is Not Affected by Season
Source: Front Cell Infect Microbiol. 2019 Dec 19;9:431. doi: 10.3389/fcimb.2019.00431 (PMC6930885; doi:10.3389/fcimb.2019.00431)
Supplement: Supplementary file 1 [file Table_1.docx]

***Supplementary Material***

**Table of Contents**

[Section 1 – Seasonal treatment of the 4-month-old nymphs 2](#_Toc20209792)

[Section 2 – Effect of tick age and tick seasonal treatment on the prevalence of *B. afzelii* infection 2](#_Toc20209793)

[Section 3 – Effect of tick age and tick seasonal treatment on the *B. afzelii* spirochete load 3](#_Toc20209794)

[Section 4 – Infection status of the mouse versus infection status of the tick 4](#_Toc20209795)

[Section 5 – Effect of competition between strains on the strain-specific transmission to immature *I. ricinus* ticks 5](#_Toc20209796)

[Section 6 – Effect of competition between strains on the strain-specific spirochete load in immature *I. ricinus* ticks 6](#_Toc20209797)

[Section 7 – Model selection using the Akaike information criterion (AIC) 7](#_Toc20209798)

[Section 8 – Post-hoc tests 9](#_Toc20209799)

**Section 1 – Seasonal treatment of the 4-month-old nymphs:**

For the natural winter treatment, the nymphs were kept in a plastic box (30 cm x 23 cm x10 cm) that was buried in the soil at a depth of 10 cm in a forest in the botanical garden of Neuchâtel (47°00’02.2”N, 6°56’15.1”E) for a period of three months (22 January 2016 to 15 April 2016). The box contained three button logs that measured the temperature every 30 minutes. Over the three-month period that the ticks were buried underground, the mean daily average, mean daily maximum, and mean daily minimum temperatures were respectively 6.44°C (range = 3.72°C – 10.50°C), 6.63°C (range = 3.79°C – 10.55°C), and 6.29°C (range = 3.58°C – 10.36°C; Fig. S1).


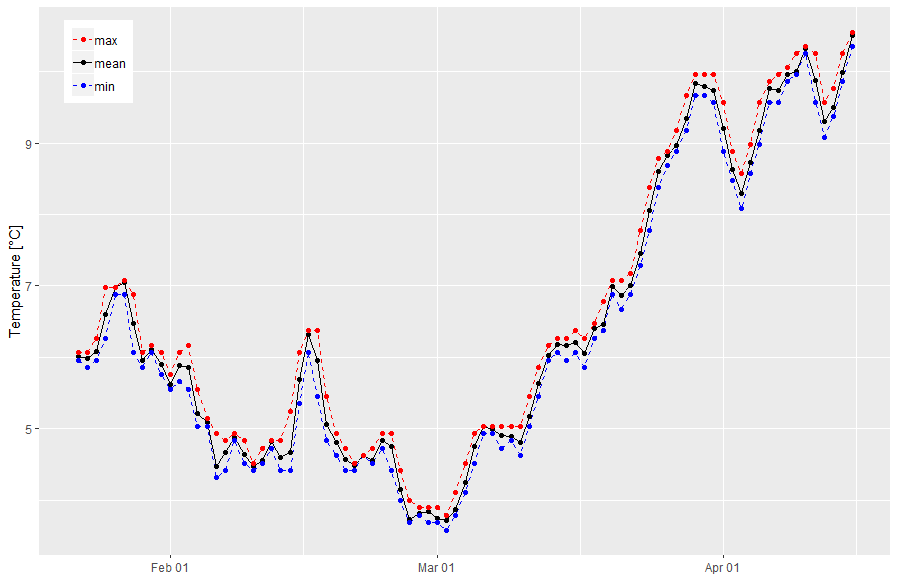


**Figure S1.** The daily average temperatures (black line), daily maximum temperatures (red line), and daily minimum (blue line) temperatures are shown for the soil at a depth of 10 cm at the Botanical Garden of Neuchâtel over a period of three months (from 22 January 2016 to 15 April 2016). Temperature was measured by temperature buttons that were placed in boxes that were buried underground. The *I. ricinus* nymphs in the natural winter treatment were exposed to this temperature treatment.

**Section 2 – Effect of tick age and tick seasonal treatment on the prevalence of *B. afzelii* infection**

We wanted to test whether tick age (larva, 1-month-old nymph, and 4-month old nymph) and seasonal treatment of the 4-month-old nymphs (phytotron, fridge, and underground) influenced tick infection status. Model comparison showed that the model with 5 treatments could be collapsed into a model with three tick ages with no loss of information (LLR test of treatment vs age: Δ df = 2, Δ dev = 2.236, p = 0.327). Similarly, when the analysis was restricted to the subset of 4-month-old nymphs, there was no effect of seasonal treatment on infection prevalence (LLR test of 4-month-old vs null: Δ df = 2, Δ dev = 2.254 p = 0.324). The rest of the statistical analysis is presented in the main manuscript in the section titled “Effect of tick age and tick seasonal treatment on the prevalence of *B. afzelii* infection”.

**Section 3 – Effect of tick age and tick seasonal treatment on the *B. afzelii* spirochete load**

We wanted to test whether tick age (larva, 1-month-old nymph, and 4-month old nymph) and seasonal treatment of the 4-month-old nymphs (phytotron, fridge, and underground) influenced the tick spirochete load. We therefore created a boxplot that shows the tick spirochete load as a function of these five groups of ticks (Figure S2). This boxplot shows that tick age has an important effect on tick spirochete load. In contrast, the seasonal treatment did not affect the spirochete loads of 4-month-old nymphs. Model comparison showed that the model with 5 treatments could be collapsed into a model with three tick ages with no loss of information (LLR test of treatment vs age: Δ df = 2, Δ dev = 2.369, p = 0.306). Similarly, when the analysis was restricted to the subset of 4-month-old nymphs, there was no significant effect of the seasonal treatment on the spirochete load (LLR test of 4-month-old vs null: Δ df = 2, Δ dev = 2.866 p = 0.239). The rest of the statistical analysis is presented in the main manuscript in the section titled “Effect of tick age and tick seasonal treatment on the *B. afzelii* spirochete load”.


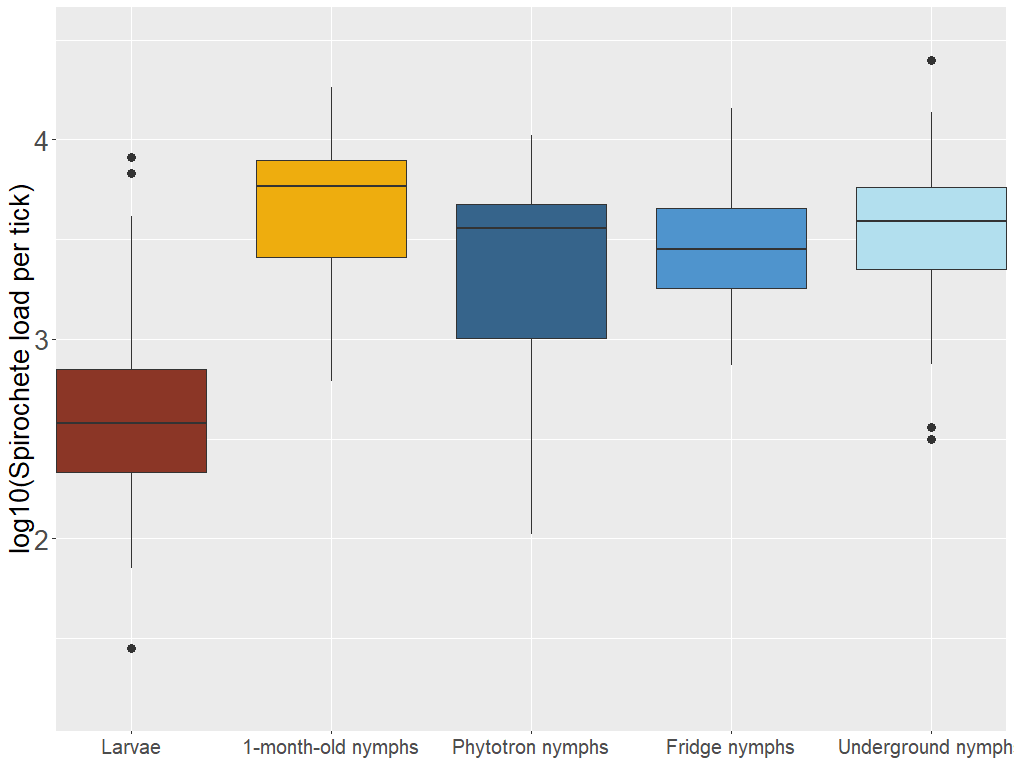


**Figure S2.** The *B. afzelii* spirochete loads inside the ticks are shown for each of the five treatments: engorged larvae, 1-month-old nymphs, 4-month-old phytotron nymphs, 4-month-old fridge nymphs, and 4-month-old underground nymphs. The spirochete loads were log10-transformed to normalize the residuals. The three seasonal treatments (phytotron, fridge, underground) had no effect on the spirochete load of the 4-month-old nymphs. Each data point represents the mean for the ticks sampled from the same mouse. The total number of ticks for each of the 5 treatments are as follows: larvae (n = 142), 1-month-old nymphs (n = 301), 4-month-old phytotron nymphs (n = 119), 4-month-old fridge nymphs (n = 120), and 4-month-old underground nymphs (n = 118). Shown are the medians (black line), the 25^th^ and 75^th^ percentiles (edges of the box), the minimum and maximum values (whiskers), and the outliers (solid circles).

**Section 4 – Infection status of the mouse versus infection status of the tick**

A co-infected mouse can produce ticks infected with strain Fin-Jyv-A3, ticks infected with strain NE4049, and ticks co-infected with both strains. In contrast, a mouse infected with a single strain can only produce ticks infected with that particular strain. Thus, for the statistical analyses of the strain-specific spirochete load (SSSL), the co-infection status of each tick can be decided using either the co-infection status of the mouse that produced the tick, or the co-infection status of the tick. The problem with analysing the SSSL in the tick (response variable) as a function of the co-infection status of the tick (explanatory variable) is that both of these variables were determined using the same qPCR protocol. Using the same data to generate both the response variable and the explanatory variable is likely to produce biased results and should be avoided. In contrast, the co-infection status of the mouse was determined using six mouse tissue samples (unpublished data) and all of the ticks that fed on that mouse. Thus, from a philosophy of science perspective, we should use mouse co-infection status rather than tick co-infection status to model the SSSL in the tick.

We can also use a model selection approach to determine which of these two explanatory variables, mouse co-infection status or tick co-infection status, is better for explaining variation in the SSSL in the tick. We therefore analysed the SSSL in the tick as a function of mouse co-infection status, tick co-infection status, strain, and tick age using linear mixed effects models (LMMs). The SSSL in the ticks were log10-transformed to normalize the residuals. All the models were compared using the Akaike information criterion (AIC); the models with the lowest AIC score are considered the best models. Interestingly, the best models used mouse co-infection status rather than tick co-infection status (Table S1). For this reason, the statistical analyses in the main manuscript used mouse co-infection status rather than tick co-infection status.

**Table S1.** Model selection results are shown for the linear mixed effects models of the log10-transformed strain-specific spirochete loads (SSSL) in the ticks. The models are ranked according to their AIC score. The explanatory variables were mouse co-infection status (M), tick co-infection status (T), strain (S), tick age (A), and their interactions. Shown for each model are the model rank (Rank), fixed effects structure of the model, model degrees of freedom (Df), log-likelihood (logLik), Akaike information criterion (AIC), difference in the AIC value from the top model (ΔAIC), model weight (Weight1; expressed as a %), and cumulative weight (Weight2; expressed as a %).

| **Rank** | **Fixed effects structure** | **Df** | **logLik** | **AIC** | **ΔAIC** | **Weight1** | **Weight2** |
| --- | --- | --- | --- | --- | --- | --- | --- |
| 1 | SSSL ~ M:S+A+M+S | 8 | -502.7 | 1021.8 | 0.0 | 22.1 | 22.1 |
| 2 | SSSL ~ A+M+S | 7 | -504.2 | 1022.5 | 0.8 | 14.9 | 37.0 |
| 3 | SSSL ~ A:S+M:S+A+M+S | 10 | -501.1 | 1022.6 | 0.8 | 14.7 | 51.7 |
| 4 | SSSL ~ A:S+A+M+S | 9 | -502.4 | 1023.2 | 1.4 | 10.7 | 62.4 |
| 5 | SSSL ~ A+M | 6 | -506.1 | 1024.4 | 2.7 | 5.8 | 68.2 |
| 6 | SSSL ~ A:T+A+T+S | 9 | -503.3 | 1025.0 | 3.3 | 4.3 | 72.4 |
| 7 | SSSL ~ A:M+M:S+A+M+S | 10 | -502.3 | 1025.0 | 3.3 | 4.3 | 76.7 |
| 8 | SSSL ~ A:T+A:S+A+T+S | 11 | -501.5 | 1025.6 | 3.8 | 3.2 | 79.9 |
| 9 | SSSL ~ A+T+S | 7 | -505.7 | 1025.7 | 3.9 | 3.1 | 83.0 |
| 10 | SSSL ~ A:M+A+M+S | 9 | -503.7 | 1025.9 | 4.1 | 2.9 | 85.9 |
| 11 | SSSL ~ A:S+A+T+S | 9 | -503.9 | 1026.1 | 4.4 | 2.5 | 88.4 |
| 12 | SSSL ~ A:M+A:S+M:S+A+M+S | 12 | -500.8 | 1026.3 | 4.5 | 2.3 | 90.7 |
| 13 | SSSL ~ A:M+A:S+A+M+S | 11 | -502.2 | 1026.9 | 5.2 | 1.7 | 92.3 |
| 14 | SSSL ~ A:T+T:S+A+T+S | 10 | -503.3 | 1027.1 | 5.3 | 1.5 | 93.9 |
| 15 | SSSL ~ A:M:S+A:M+A:S+M:S+A+M+S | 14 | -499.4 | 1027.6 | 5.8 | 1.2 | 95.1 |
| 16 | SSSL ~ A:T+A:S+T:S+A+T+S | 12 | -501.5 | 1027.7 | 5.9 | 1.1 | 96.2 |
| 17 | SSSL ~ T:S+A+T+S | 8 | -505.7 | 1027.7 | 6.0 | 1.1 | 97.3 |
| 18 | SSSL ~ A+T | 6 | -508.0 | 1028.1 | 6.3 | 0.9 | 98.2 |
| 19 | SSSL ~ A:S+T:S+A+T+S | 10 | -503.9 | 1028.2 | 6.5 | 0.9 | 99.1 |
| 20 | SSSL ~ A:T:S+A:T+A:S+T:S+A+T+S | 14 | -500.9 | 1030.7 | 8.9 | 0.3 | 99.4 |
| 21 | SSSL ~ A+S | 6 | -509.5 | 1031.1 | 9.3 | 0.2 | 99.6 |
| 22 | SSSL ~ A+S | 6 | -509.5 | 1031.1 | 9.3 | 0.2 | 99.8 |
| 23 | SSSL ~ A | 5 | -511.2 | 1032.5 | 10.8 | 0.1 | 99.9 |
| 24 | SSSL ~ A | 5 | -511.2 | 1032.5 | 10.8 | 0.1 | 100.0 |
| 25 | SSSL ~ M+S | 5 | -547.4 | 1104.8 | 83.1 | 0.0 | 100.0 |
| 26 | SSSL ~ M | 4 | -548.7 | 1105.4 | 83.7 | 0.0 | 100.0 |
| 27 | SSSL ~ T+S | 5 | -551.3 | 1112.7 | 90.9 | 0.0 | 100.0 |
| 28 | SSSL ~ T | 4 | -552.8 | 1113.6 | 91.9 | 0.0 | 100.0 |
| 29 | SSSL ~ S | 4 | -552.9 | 1113.8 | 92.1 | 0.0 | 100.0 |
| 30 | SSSL ~ S | 4 | -552.9 | 1113.8 | 92.1 | 0.0 | 100.0 |
| 31 | SSSL ~ 1 | 3 | -554.1 | 1114.2 | 92.5 | 0.0 | 100.0 |
| 32 | SSSL ~ 1 | 3 | -554.1 | 1114.2 | 92.5 | 0.0 | 100.0 |

The acronyms for the explanatory variables are as follows: SSSL = strain-specific spirochete load in the tick, M = mouse co-infection status, T = tick co-infection status, S = strain, A = tick age.

**Section 5 – Effect of competition between strains on the strain-specific transmission to immature *I. ricinus* ticks**

We wanted to test the effects of competition, strain, tick stage, and tick seasonal treatment on the strain-specific transmission from the rodent host to *I. ricinus* ticks. Model comparison showed that there was no effect of the seasonal treatment on the strain-specific prevalence in the 4-month-old nymphs and that we were justified in combining the 4-month-old nymphs into a single group (LLR test of treatment vs age: Δ df = 8, Δ dev = 5.271, p = 0.728). Further model comparison showed that the three tick ages could be collapsed into two tick stages with no loss of information (LLR test of stage vs age: Δ df = 4, Δ dev = 4.846, p = 0.304). Thus, the strain-specific prevalence was analysed as a function of three fixed factors: strain, competition, tick stage, and their interactions.

A classic step-wise model simplification approach using log-likelihood ratio (LLR) tests found that the three-way interaction (LLR: Δ df = 1, Δ dev = 3.355, p = 0.067) and the two-way interaction between strain and tick stage (LLR: Δ df = 1, Δ dev = 0.076, p = 0.783) were not significant. In contrast, the two-way interactions between competition and tick stage (LLR: Δ df = 1, Δ dev = 6.736, p = 0.0094), and between strain and competition (LLR: Δ df = 1, Δ dev = 4.760, p = 0.029) were significant. In summary, the best model included the following fixed effects: strain, competition, tick stage, competition:tick stage interaction, and strain:competition interaction. The parameter estimates for this model are shown in Table S2. The rest of the statistical analysis is presented in the main manuscript in the section titled “Effect of competition between strains on the strain-specific transmission to immature *I. ricinus* ticks”. The AIC-based model selection approach converged on the same model (see model 1 in Table S4 in section 7).

**Table S2.** The parameter estimates are shown for the best GLMM model from the step-wise regression analysis of the strain-specific prevalence in the immature *I. ricinus* ticks. The best model included the main effect of strain (Fin-Jyv-A3, NE4049), the main effect of competition (no, yes), the main effect of tick stage (larvae, nymphs), the interaction between tick stage and competition, and the interaction between strain and competition. The parameters include an intercept (defined as strain Fin-Jyv-A3 in a larva with no competition), main effects (expressed as the difference between the levels of a factor), and interactions. Shown are the parameter estimates (Estimates), standard errors (SE), z-values, and the associated p-values (p).

| Factor | Estimates | SE | z-value | p |
| --- | --- | --- | --- | --- |
| Intercept: Fin-Jyv-A3/Larvae/No | -0.479 | 0.455 | -1.053 | 0.292 |
| Strain: NE4049 - Fin-Jyv-A3 | -1.177 | 0.581 | -2.026 | 0.043 |
| Competition: Yes - No | -1.133 | 0.707 | -1.601 | 0.109 |
| Tick stage: Nymph - Larva | 3.016 | 0.348 | 8.674 | < 0.001 |
| Interaction: Strain:Competition | 1.911 | 0.837 | 2.282 | 0.023 |
| Interaction: Competition:Tick stage | -1.301 | 0.500 | -2.602 | 0.009 |
|  |  |  |  |  |

**
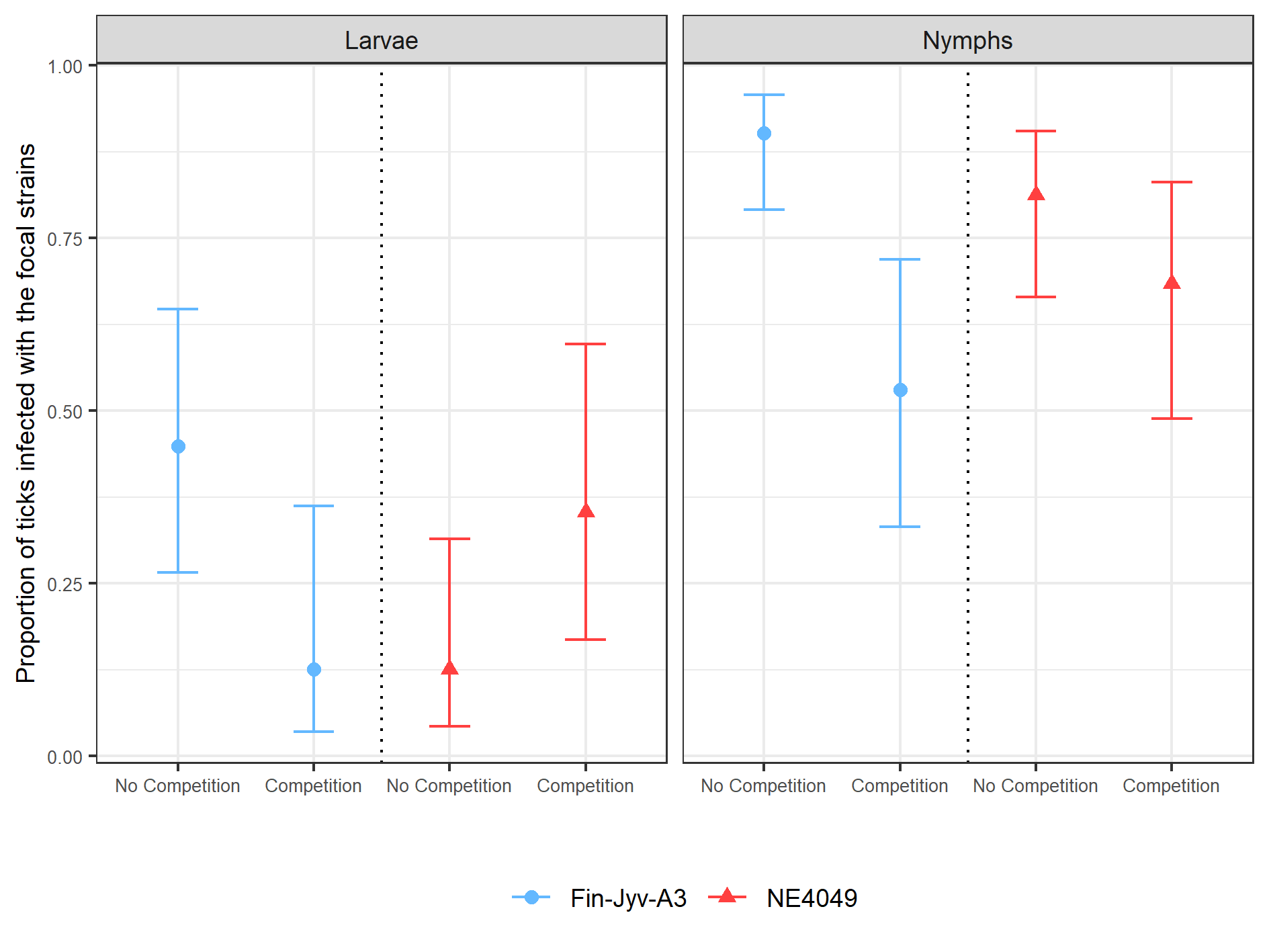
**

**Figure S3.** The proportion of immature *I. ricinus* ticks infected with *B. afzelii* is shown as a function of three factors: (1) tick age (engorged larvae, nymphs), (2) strain (Fin-Jyv-A3 in blue and NE4049 in red), and (3) competition (no competition versus competition). The proportion of infected ticks is an estimate of host-to-tick transmission. The 1-month-old nymphs and 4-month-old nymphs were combined because our statistical analysis found no difference between these two types of nymphs. According to the parameter estimates of our statistical analysis, competition between strains in co-infected mice reduced host-to-tick transmission of strain Fin-Jyv-A3 to engorged larvae and nymphs and reduced the host-to-tick transmission of strain NE4049 to nymphs. In contrast, competition between strains in co-infected mice did not influence the host-to-tick transmission of strain NE4049 to engorged larvae. The graph shows the means and the 95% confidence intervals.

**Section 6 – Effect of competition between strains on the strain-specific spirochete load in immature *I. ricinus* ticks**

We wanted to test the effects of competition, strain, tick stage, and tick seasonal treatment on the strain-specific spirochete load in the subset of *I. ricinus* ticks infected with *B. afzelii*. Model comparison showed that there was no effect of the seasonal treatment on the *B. afzelii* spirochete load in the 4-month-old nymphs and that we were justified in combining the 4-month-old nymphs into a single group (LLR test of treatment vs age: Δ df = 8, Δ dev = 6.016, p = 0.645). No further model simplification was justified because the 1-month-old nymphs and 4-month-old nymphs were significantly different (LLR test of age vs stage: Δ df = 4, Δ dev = 13.827, p = 0.008). Thus, the strain-specific spirochete load was analysed as a function of three fixed factors: strain, competition, tick age, and their interactions.

The log10-transformed strain-specific spirochete load was analysed as a function of three fixed factors: strain, competition, tick age, and their interactions. A classic step-wise model simplification approach using LLR tests found that the three-way interaction was not significant (LLR: Δ df = 2, Δ dev = 2.933, p = 0.231) and it was removed from the model. None of the two-way interactions were significant (LLR test of competition:age interaction: Δ df = 2, Δ dev = 0.468, p = 0.792, LLR test of strain:age interaction: Δ df = 2, Δ dev = 2.942, p = 0.230, LLR test of strain:competition interaction: Δ df = 1, Δ dev = 2.725, p = 0.099) and they were removed from the model. In summary, the best model included the main effects of strain, competition, and tick stage. The parameter estimates for this model are shown in Table S3. The rest of the statistical analysis is presented in the main manuscript in the section titled “Effect of competition between strains on the strain-specific spirochete load in immature *I. ricinus* ticks”. The AIC-based model selection approach converged on the same model (see model 2 in Table S5 in section 7).

**Table S3.** The parameter estimates are shown for the best LMM model from the step-wise regression analysis of the strain-specific spirochete load in the immature *I. ricinus* ticks. The best model included the main effects of strain (Fin-Jyv-A3, NE4049), competition (no, yes), and tick age (larva, 1-month-old nymph, 4-month-old nymph). The parameters include an intercept (defined as strain Fin-Jyv-A3 in a larva with no competition) and main effects (expressed as the difference between the levels of a factor). Shown are the parameter estimates (Estimates), standard errors (SE), degrees of freedom (df), t-values, and the associated p-values (p).

| Parameter | Estimates | SE | df | t-value | p |
| --- | --- | --- | --- | --- | --- |
| Intercept: Fin-Jyv-A3/Larva/No | 2.681 | 0.117 | 164.619 | 23.000 | < 0.001 |
| Strain: NE4049 - Fin-Jyv-A3 | -0.170 | 0.084 | 24.825 | -2.038 | 0.052 |
| Competition: Yes - No | -0.292 | 0.085 | 25.047 | -3.454 | 0.002 |
| Tick age: 1-month nymph - larva | 1.080 | 0.110 | 495.073 | 9.797 | < 0.001 |
| Tick age: 4-month nymph - larva | 0.887 | 0.111 | 507.922 | 7.969 | < 0.001 |


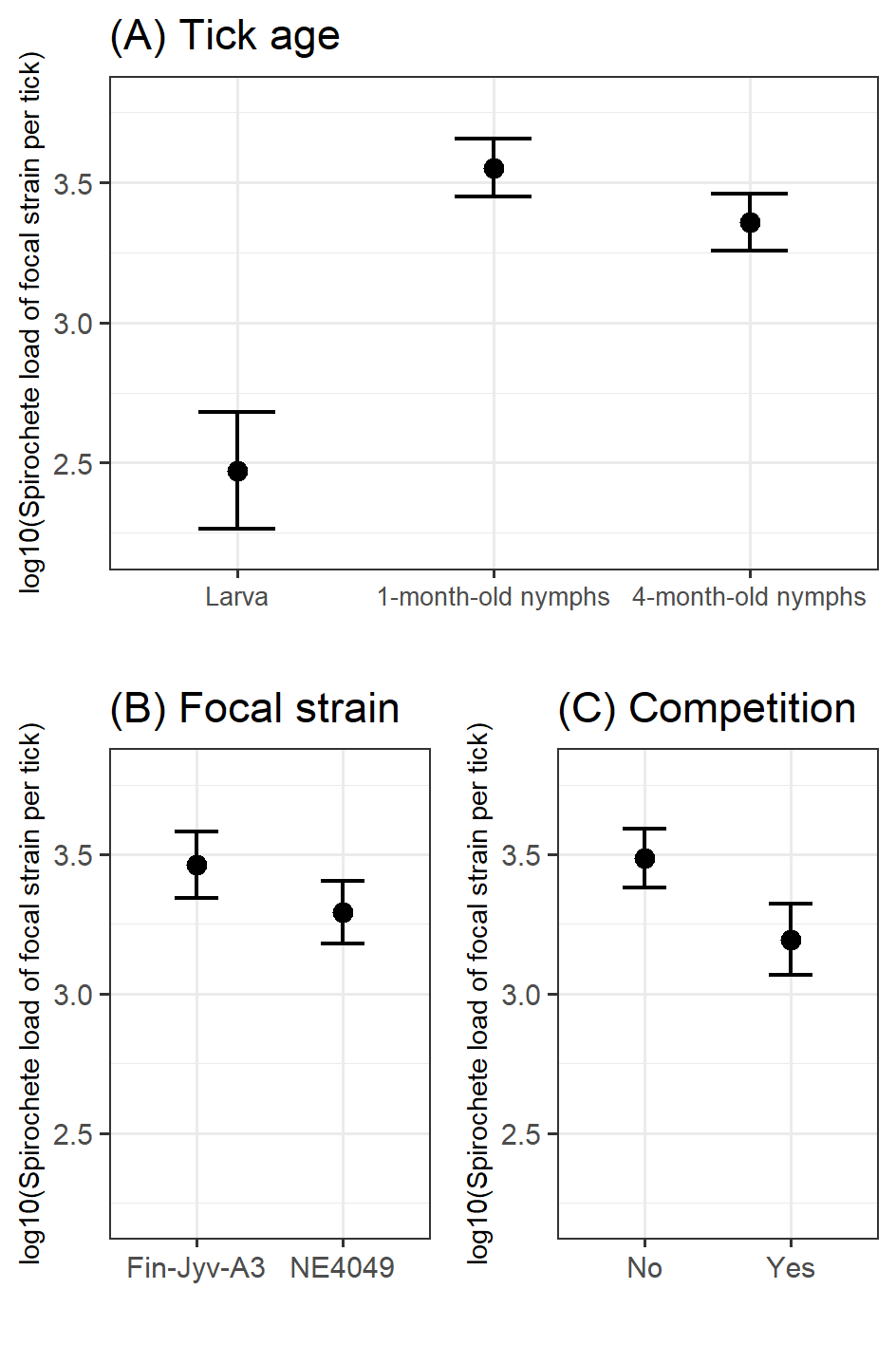


**Figure S4.** The *B. afzelii* spirochete loads in immature *I. ricinus* ticks are shown as a function of three factors: (A) tick age (engorged larvae, 1-month-old nymphs, and 4-month-old nymphs), (B) strain, and (C) competition. The spirochete loads were log10-transformed to normalize the residuals. According to the parameter estimates of our statistical analysis, competition between strains reduced the spirochete loads of both strains in all three types of ticks. The graph shows the means and the 95% confidence intervals.

**Section 7 – Model selection using the Akaike information criterion (AIC)**

For the analysis of the strain-specific prevalence and the strain-specific spirochete load in immature *I. ricinus* ticks, we used a classic step-wise model simplification approach to identify the best model (see section 5 and section 6, respectively). Specifically, we compared nested models using log-likelihood ratio (LLR) tests and removed non-significant interaction terms until we obtained a model where all of the remaining factors (or their interactions) were statistically significant. We also used AIC-based model selection to determine whether a different approach would identify the same model as being the best one.

In the analysis of the strain-specific prevalence in immature *I. ricinus* ticks, a classic stepwise model simplification approach using LRR tests found that the best model contained the three fixed factors: strain, competition, and tick stage and the interactions between strain and competition, and between competition and tick stage (see section 5). The AIC-based model selection approach also identified this model as the best (see model 1 in Table S4). In the analysis of the strain-specific spirochete load in immature *I. ricinus* ticks, a classic stepwise model simplification approach using LRR tests found that the best model contained the three fixed factors: strain, competition, and tick age (see section 6). The AIC-based model selection approach also identified this model as the best (model 2 in Table S5). In Table S5, the second-place model is actually the best model because it has fewer parameters than the first-place model and because ΔAIC < 1 (i.e. the simpler second-place model is within 1 AIC unit of the more complex first-place model).

**Table S4.** Model selection results are shown for the generalized linear mixed effects models of the strain-specific transmission (SST) to immature *I. ricinus* ticks. The models are ranked according to their AIC score. The explanatory variables were competition (C), strain (S), and tick stage (T). Shown for each model are the model rank (Rank), fixed effects structure of the model, model degrees of freedom (Df), log-likelihood (logLik), Akaike information criterion (AIC), difference in the AIC value from the top model (ΔAIC), model weight (Weight1; expressed as a %), and cumulative weight (Weight2; expressed as a %).

| **Rank** | **Fixed effects structure** | **Df** | **logLik** | **AIC** | **ΔAIC** | **Weight1** | **Weight2** |
| --- | --- | --- | --- | --- | --- | --- | --- |
| 1 | SST ~ S+C+T+S:C+T:C | 7 | -414.2 | 842.6 | 0.0 | 37.2 | 37.2 |
| 2 | SST ~ T+C+S+T:C+T:S+C:S+T:C:S | 9 | -412.5 | 843.3 | 0.7 | 26.8 | 64.0 |
| 3 | SST ~ S+C+T+S:C+T:S+T:C | 8 | -414.2 | 844.6 | 2.0 | 13.9 | 77.9 |
| 4 | SST ~ S+C+T+T:C | 6 | -416.7 | 845.5 | 2.9 | 8.9 | 86.8 |
| 5 | SST ~ S+C+T+S:C | 6 | -417.6 | 847.2 | 4.6 | 3.7 | 90.5 |
| 6 | SST ~ S+C+T+T:S+T:C | 7 | -416.6 | 847.3 | 4.7 | 3.6 | 94.1 |
| 7 | SST ~ C+T | 4 | -419.9 | 847.9 | 5.3 | 2.7 | 96.7 |
| 8 | SST ~ S+C+T+S:C+T:S | 7 | -417.6 | 849.3 | 6.7 | 1.3 | 98.1 |
| 9 | SST ~ S+C+T | 5 | -419.8 | 849.7 | 7.1 | 1.1 | 99.1 |
| 10 | SST ~ S+C+T+T:S | 6 | -419.8 | 851.7 | 9.0 | 0.4 | 99.5 |
| 11 | SST ~ T | 3 | -423.0 | 852.1 | 9.5 | 0.3 | 99.9 |
| 12 | SST ~ S+T | 4 | -422.9 | 853.9 | 11.3 | 0.1 | 100.0 |
| 13 | SST ~ C | 3 | -478.2 | 962.3 | 119.7 | 0.0 | 100.0 |
| 14 | SST ~ S+C | 4 | -478.2 | 964.4 | 121.8 | 0.0 | 100.0 |
| 15 | SST ~ 1 | 2 | -480.7 | 965.4 | 122.8 | 0.0 | 100.0 |
| 16 | SST ~ S | 3 | -480.7 | 967.4 | 124.8 | 0.0 | 100.0 |

The acronyms for the response and explanatory variables are as follows: SST = strain-specific transmission, C = competition, S = strain, and T = tick stage.

**Table S5.** Model selection results are shown for the linear mixed effects models of the log10-transformed strain-specific spirochete loads (SSSL). The models are ranked according to their AIC score. The explanatory variables were competition (C), strain (S), and tick age (A). Shown for each model are the model rank (Rank), fixed effects structure of the model, model degrees of freedom (Df), log-likelihood (logLik), Akaike information criterion (AIC), difference in the AIC value from the top model (ΔAIC), model weight (Weight1; expressed as a %), and cumulative weight (Weight2; expressed as a %).

| **Rank** | **Fixed effects structure** | **Df** | **logLik** | **AIC** | **ΔAIC** | **Weight1** | **Weight2** |
| --- | --- | --- | --- | --- | --- | --- | --- |
| 1 | SSSL ~ A+C+S+C:S | 8 | -502.7 | 1021.8 | 0.0 | 27.3 | 27.3 |
| 2 | SSSL ~ A+C+S | 7 | -504.2 | 1022.5 | 0.7 | 18.5 | 45.8 |
| 3 | SSSL ~ A+C+S+A:S+C:S | 10 | -501.1 | 1022.6 | 0.8 | 18.2 | 64.0 |
| 4 | SSSL ~ A+C+S+A:S | 9 | -502.4 | 1023.2 | 1.4 | 13.3 | 77.3 |
| 5 | SSSL ~ A+C | 6 | -506.1 | 1024.4 | 2.6 | 7.2 | 84.5 |
| 6 | SSSL ~ A+C+S+A:C+C:S | 10 | -502.3 | 1025.1 | 3.3 | 5.3 | 89.8 |
| 7 | SSSL ~ A+C+S+A:C | 9 | -503.8 | 1025.9 | 4.1 | 3.5 | 93.3 |
| 8 | SSSL ~ A+C+S+A:C+A:S+C:S | 12 | -500.8 | 1026.3 | 4.5 | 2.8 | 96.1 |
| 9 | SSSL ~ A+C+S+A:C+A:S | 11 | -502.2 | 1026.9 | 5.1 | 2.1 | 98.2 |
| 10 | SSSL ~ A+C+S+A:C+A:S+C:S+A:C:S | 14 | -499.4 | 1027.6 | 5.8 | 1.5 | 99.7 |
| 11 | SSSL ~ A+S | 6 | -509.5 | 1031.1 | 9.3 | 0.3 | 99.9 |
| 12 | SSSL ~ A | 5 | -511.2 | 1032.5 | 10.7 | 0.1 | 100.0 |
| 13 | SSSL ~ C+S | 5 | -547.4 | 1104.8 | 83.0 | 0.0 | 100.0 |
| 14 | SSSL ~ C | 4 | -548.7 | 1105.5 | 83.7 | 0.0 | 100.0 |
| 15 | SSSL ~ S | 4 | -552.9 | 1113.8 | 92.0 | 0.0 | 100.0 |
| 16 | SSSL ~ 1 | 3 | -554.1 | 1114.2 | 92.4 | 0.0 | 100.0 |

The acronyms for the response and explanatory variables are as follows: SSSL = log10-transformed strain-specific spirochete loads, C = competition, S = strain, and A = tick age.

**Section 8 – Post-hoc tests**

Pairwise comparisons for GLMMs and LMMs were done using the functions ‘emmeans’, ‘contrast’, and ‘pairs’ in the emmeans package. Tables S6, S7, and S8 show the results of the post-hoc tests.

**Table S6. Pairwise comparisons of *B. afzelii* infection prevalence among tick ages:** The *B. afzelii* infection prevalence was modelled as a function of tick age and seasonal treatment using GLMMs. Tick age was significant, and we therefore used post-hoc pairwise comparisons to determine which of the three tick ages (engorged larvae, 1-month-old nymphs, and 4-months-old nymphs) were significantly different from each other. For each pairwise comparison, the z-ratio and the associated p-values (p) are shown. These p-values are presented in the main manuscript in the section titled “Effect of tick age and tick seasonal treatment on the prevalence of *B. afzelii* infection”.

| qPCR | Pairwise comparison | z-ratio | p |
| --- | --- | --- | --- |
| *flagellin* and *ospC* | Larva – 1-month-old nymph | -10.146 | <0.0001 |
| *flagellin* and *ospC* | Larva – 4-month-old nymph | -9.432 | <0.0001 |
| *flagellin* and *ospC* | 1-month-old nymph – 4-month old nymph | 1.590 | 0.249 |

**Table S7. Pairwise comparisons of total *B. afzelii* spirochete load among tick ages:** The total *B. afzelii* spirochete load for the subset of infected ticks was modelled as a function of tick age and seasonal treatment using LMMs. Tick age was significant, and we therefore used post-hoc pairwise comparisons to determine which of the three tick ages (engorged larvae, 1-month-old nymphs, and 4-months-old nymphs) were significantly different from each other. For each pairwise comparison, the degrees of freedom (df), the z-ratio, and the associated p-values (p) are shown. These p-values are presented in the main manuscript in the section titled “Effect of tick age and tick seasonal treatment on the *B. afzelii* spirochete load”.

| qPCR | Pairwise comparison | df | t-ratio | p |
| --- | --- | --- | --- | --- |
| *flagellin* | Larva – 1-month-old nymph | 593.13 | -11.824 | <0.0001 |
| *flagellin* | Larva – 4-month-old nymph | 608.00 | -9.346 | <0.0001 |
| *flagellin* | 1-month-old nymph – 4-month old nymph | 608.86 | 4.065 | 0.0002 |

**Table S8. Pairwise comparisons of strain-specific spirochete load among tick ages:** The strain-specific spirochete load for the subset of infected ticks was modelled as a function of strain, competition, and tick age using LMMs. Tick age was significant, and we therefore used post-hoc pairwise comparisons to determine which of the three tick ages (engorged larvae, 1-month-old nymphs, and 4-months-old nymphs) were significantly different from each other. For each pairwise comparison, the degrees of freedom (df), the z-ratio, and the associated p-values (p) are shown. These p-values are presented in the main manuscript in the section titled “Effect of competition between strains on the strain-specific spirochete load in immature *I. ricinus* ticks”.

| qPCR | Pairwise comparison | df | t-ratio | p |
| --- | --- | --- | --- | --- |
| *ospC* | Larva – 1-month-old nymph | 499.17 | -9.754 | <0.0001 |
| *ospC* | Larva – 4-month-old nymph | 513.33 | -7.899 | <0.0001 |
| *ospC* | 1-month-old nymph – 4-month old nymph | 513.28 | 3.150 | <0.0046 |
